# The role of bacterial metabolism in human gut colonization

**DOI:** 10.1007/s10123-024-00550-6

**Published:** 2024-06-28

**Authors:** Ada Muñoz-Cazalla, Ignacio de Quinto, Laura Álvaro-Llorente, Jerónimo Rodríguez-Beltrán, Cristina Herencias

**Affiliations:** 1 Servicio de Microbiología, Instituto Ramón y Cajal de Investigación Sanitaria (IRYCIS), Hospital Universitario Ramón y Cajal, Madrid, Spain; 2 Centro de Investigación Biomédica en Red de Enfermedades Infecciosas-CIBERINFEC, Instituto de Salud Carlos III, Madrid, Spain

**Keywords:** Antibiotic resistance, Genome-scale metabolic models, Gut colonization, High-risk clones

## Abstract

Can we anticipate the emergence of the next pandemic antibiotic-resistant bacterial clone? Addressing such an ambitious question relies on our ability to comprehensively understand the ecological and epidemiological factors fostering the evolution of high-risk clones. Among these factors, the ability to persistently colonize and thrive in the human gut is crucial for most high-risk clones. Nonetheless, the causes and mechanisms facilitating successful gut colonization remain obscure. Here, we review recent evidence that suggests that bacterial metabolism plays a pivotal role in determining the ability of high-risk clones to colonize the human gut. Subsequently, we outline novel approaches that enable the exploration of microbial metabolism at an unprecedented scale and level of detail. A thorough understanding of the constraints and opportunities of bacterial metabolism in gut colonization will foster our ability to predict the emergence of high-risk clones and take appropriate containment strategies.

## Antimicrobial resistance

 Antimicrobial resistance (AR) poses a grave threat to modern medicine and jeopardizes the effectiveness of global public health responses to infectious diseases (O’Neill 2016; World Health Organization 2017; Centers for Disease Control and Prevention (U.S.) 2019; European Centre for Disease Prevention and Control. 2019; Murray et al. 2022). The overuse and misuse of antibiotics have led to a significant increase in the prevalence of antibiotic-resistant bacteria. As a result, many people now die each year from infections that were previously treatable (O’Neill 2016; Murray et al. 2022). Current estimations predict that AR is responsible for 700,000 deaths annually and that, by 2050, AR infections could become the leading cause of death worldwide, causing up to 10 million deaths per year (O’Neill 2016; Murray et al. 2022). The magnitude of the AR crisis has been recognized by health authorities, such as the European and American Centers for Disease Prevention and Control (ECDC, CDC) (Centers for Disease Control and Prevention (U.S.) 2019; European Centre for Disease Prevention and Control. 2019). In addition, the quadripartite World Health Organization (WHO)-Food and Agriculture Organization of the United Nations (FAO)-World Organization for Animal Health (OIE)-United Nations Environmental Programme (UNEP) recognizes that critical efforts are required to reverse current dynamics and regain control over antibiotic-resistant bacteria (United Nations Environment Programme 2023; World Health Organization (WHO), Food and Agriculture Organization (FAO), World Organisation for Animal Health (WOAH), UN Environment Programme (UNEP) 2022).

Arguably, one of the main drivers of this significant health problem is the emergence and global dissemination of epidemiologically successful drug-resistant clones. These high-risk clones have acquired adaptive traits that increase their pathogenicity and survival, including the acquisition of AR. Due to their unique combination of virulence, metabolic, and AR genes, these clones pose a significant risk to human health, spreading uncontrollably in the community and hospital settings and different One Health sectors. Additionally, they contribute to the global spread of AR by transmitting genetic platforms such as transposons or plasmids to unrelated bacteria via horizontal gene transfer (Woodford et al. 2011).

Among these high-risk clones, multidrug-resistant pathogenic *Escherichia coli* has been categorized as an “urgent threat” by the CDC and a “critical priority” by the WHO. *E. coli* is a Gram-negative commensal in the human gastrointestinal tract and, simultaneously, one of the most frequent pathogens. Indeed, *E. coli* is the most common cause of extra-intestinal infections, such as urinary tract infections and bloodstream infections (Pitout 2012). Extra-intestinal *E. coli* (ExPEC) high-risk clones mainly belong to the phylogenetic group B2 and, to a lesser extent, phylogenetic group D (Le Gall et al. 2007; Massot et al. 2016; Nowrouzian et al. 2019). B2 isolates exhibit considerable genome diversity, with 10 subgroups from B2-I to B2-X, and are overrepresented by specific sequence-type complexes that show a higher ability to persist and colonize the human gut (Mansouri et al. 2022). For instance, *E. coli* belonging to the sequence-type complex ST131 accounts for up to 30% of all infections caused by ExPEC globally and is responsible for 60–90% of the fluoroquinolone-resistant and 40–80% of extended-spectrum β-lactamase-producing ExPEC infections (Nicolas-Chanoine et al. 2007). Recently, new clinically relevant clonal complexes have been identified. For example, ST410 emerged as a significant global concern, becoming increasingly common in Chinese hospitals since 2017 (Ba et al. 2024).

The ability of these worrisome clonal complexes (and those of other species) to colonize the human gut is key to their ecological and epidemiological success. Therefore, understanding how they colonize and thrive within the intricate environment of the gastrointestinal tract is crucial to predict, anticipate, and mitigate their dissemination. In the following sections, we will outline the factors that facilitate bacterial colonization of the human gut and discuss experimental approaches to identify these factors.

## Ecological interactions driving a successful gut colonization

The gut microbiota is the largest bacterial population cohabiting with humans. It functions as a dynamic biological factory that evolves and adapts with the host, significantly affecting health and influencing various diseases (Minagar et al. 2023). The beneficial impact of gut microbiota on human metabolic health is extensively documented in the literature (Fan and Pedersen 2021; Liu et al. 2022). For example, the microbiota plays a crucial role in the digestion and absorption of dietary lipids, proteins, and peptides (Fan et al. 2015; Martinez-Guryn et al. 2018). However, the mechanisms employed by high-risk clones to invade and colonize the healthy microbiota remain poorly understood.

**Fig. 1:**
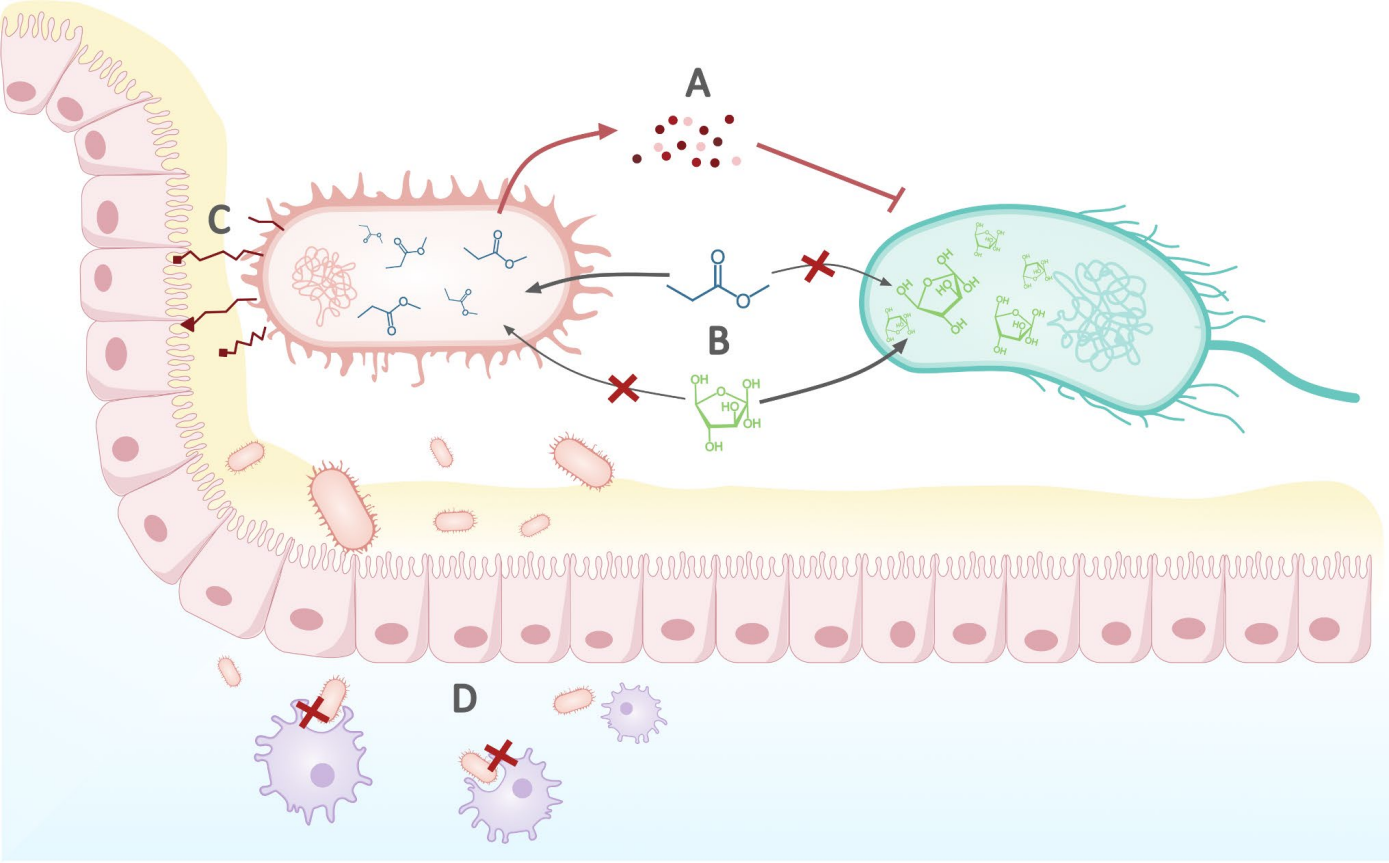
Examples of main factors driving successful gut colonization. (**A**) Pathogenic bacteria (depicted as a red cell on the left side) compete with established populations by secreting bacteriocins and colicins that inhibit the growth of resident microbiota (shown in light blue on the right side). (**B**) Consuming different carbon sources allows pathogens to thrive within the rest of the community. Commensal cells use available metabolites and generate residual byproducts (represented as green and dark blue molecules, respectively) that colonizer bacteria can use as carbon sources that otherwise would not be used. These populations can also metabolize available nutrients, establishing a competitive relationship with the pre-existing populations. (**C**) Colonization ability requires contact with the intestinal epithelial cells. Different surface characteristics, such as expressing the O-antigen, improve adhesion. (**D**) Evasion of the host immune system responses, such as escaping from phagocyte recognition (purple cells), is crucial for successful colonization

While many microbial species are regularly consumed through food ingestion, only a small proportion will successfully establish a long-lasting population in the gut (Powell et al. 2016). Successful colonization depends on several factors, including the complex ecological interactions between the resident gut microbiota and the potential colonizer bacteria (Schluter and Foster 2012; Coyte and Rakoff-Nahoum 2019). These interactions can be dynamic, ranging from resource competition to cooperative partnerships. For example, high-risk clones actively secrete toxic products such as bacteriocins, colicins, or microcins that inhibit the growth of the resident microbiota, illustrating the fierce competition that takes place within the gut microbiota (Micenková et al. 2016) (Fig. 1A). Other pathogenic bacteria outcompete gut commensals by using carbon sources that are unavailable to them. For instance, *Salmonella enterica* metabolizes propionate, ethanolamine, and ascorbate. These carbon sources are typically inaccessible to commensal bacteria, allowing *S. enterica* to colonize the gut (Asten and Dijk 2005; Harvey et al. 2011). Similarly, multidrug-resistant *E. coli* strains able to use a broad range of carbohydrates displace commensal *E. coli* from the gut, highlighting the importance of resource competition in colonization (Connor et al. 2023). In other cases, potential colonizers do not compete but can benefit from the existing microbiota. For instance, *S. enterica* serovar Typhimurium uses lactate produced by fermentative bacteria as a carbon source. In this way, *S. Typhimurium* engages in a commensalism relationship with resident bacteria, where fermenters provide a valuable resource for *S. Typhimurium* colonization (Taylor and Winter 2020) (Fig. 1B).

Beyond interbacterial ecological relationships, gut bacteria engage in a dynamic interplay with the host. Successful colonization often depends on adhering to the intestinal epithelium. This attachment can be facilitated by bacterial specialized surface structures, such as the O-antigen or *E. coli*’s type 1 fimbria, which increases interaction with the mannosylated receptors on the epithelial mucosa (Fig. 1C) (Krogfelt et al. 1990; Hölzer et al. 2009; Powell et al. 2016). Other factors, such as flagella expression, also aid in successful colonization since bacteria require flagellar mobility to cross the mucus layer and reach the surface of the epithelium (Sevrin et al. 2020).

Gut colonization also requires evading or tolerating host immune defenses. Some bacteria, like *Helicobacter pylori*, can evade host immune responses by modifying their surface antigens (e.g, lipopolysaccharide and flagellin) or by secreting immunomodulatory factors, such as nitric oxide (Fig. 1D, Lina 2014). Besides, bacteria expressing higher levels of serum survival protein (iss), a well-recognized virulence factor in ExPEC isolates, can avoid the host’s immune system. *iss* gene is necessary for capsule synthesis and facilitates avoiding phagocyte recognition (Sarowska et al. 2019; Biran et al. 2021).

## Metabolism and colonization

Beyond the ecological factors outlined above, bacterial metabolism plays a central role in gut colonization (Nogales and Garmendia 2022). Indeed, adapting and thriving in a chemically complex environment such as the gut microbiota requires a set of fine-tuned metabolic traits that translate into high bacterial fitness (i.e., competitive ability) (Bernhardt et al. 2020).

However, the high microbial diversity of the gut microbiome, encompassing a vast array of species, presents a significant challenge to understanding its metabolic complexity. From obligate aerobes to strict anaerobes, commensal bacteria exhibit a wide range of metabolic profiles. Further complicating this scenario, some species exhibit exceptional metabolic plasticity, adapting their metabolism to changing environments. For instance, *Bacillus fragilis*, a strictly anaerobic bacterium, can metabolize certain carbohydrates in the presence of nanomolar oxygen concentrations (such as those found in the colon’s crypts) thanks to the expression of the commensal colonization factor (*ccf)* genes. These genes allow *B. fragillis* to use some polysaccharides otherwise undigestible in microaerobiosis. Crucially, strains lacking *ccf* genes fail to colonize the gut effectively (Lee et al. 2013), exemplifying a case in which metabolic plasticity is key to colonization.

The commensal microbiota can actively prevent gut colonization of harmful bacteria thanks to its own metabolic processes. For instance, certain strains can limit the establishment of vancomycin-resistant *Enterococcus* (VRE) in the gut of mice treated with antibiotics. These protective strains reduce the available fructose levels in the gut, a sugar that fuels VRE growth in vivo (Djukovic et al. 2022; Isaac et al. 2022). Similarly, the commensal *Clostridium scindens* produces secondary bile acids that directly inhibit the growth and colonization of *Clostridioides difficile* (Buffie et al. 2015). Moreover, experimental evidence supports the notion that commensal bacteria use a certain degree of metabolic flexibility to outcompete phylogenetically close pathogens. For example, when *E. coli* Nissle 1917 (a probiotic non-pathogenic strain) colonizes the mice gut, and no other *E. coli* strains are present in the community, it uses ribose as a carbon source. However, when competing with other *E. coli* strains, *E. coli* Nissle alters its nutrient preferences and avoids consuming ribose, highlighting that the presence of other microorganisms (and their metabolisms) critically affects the colonization capacity of *E. coli*.

Central metabolic pathways are usually highly conserved, comprising a significant part of the core genome (Vieira et al. 2011). Several examples highlight the crucial role that central metabolism plays in the gut colonization of species like *E. coli*, the most frequent pathogen causing extraintestinal infections. For instance, *E. coli* mutants lacking phosphoglucose isomerase, a key glycolytic enzyme encoded by the *pgi* gene, display a substantial colonization defect when competing against their respective wild-type strain (Chang et al. 2004). Similarly, mutants lacking the Entner-Doudoroff pathway are defective in colonization due to their inability to effectively metabolize gluconate, the most abundant carbon source in the intestine (Sweeney et al. 1996). Interestingly, the oxidative branch of the pentose phosphate pathway is dispensable for *E. coli* colonization in mouse models. This suggests that *E. coli* may obtain the reducing power essential for sugar catabolism (i.e., nicotinamide adenine dinucleotide phosphate (NADPH)) using an alternative, unknown mechanism (Chang et al. 2004).

Altogether, this evidence suggests that efficient bacterial colonization of the gut is likely determined by (i) the availability of nutrients that bacteria can use (metabolic range), (ii) the ability to use these available carbon sources more efficiently than the rest of the members in the community (metabolic efficiency), and (iii) the ability to adapt their metabolism to the rest of the community members (metabolic flexibility). Therefore, measuring, understanding, and predicting bacterial metabolism in the gut is crucial to anticipate the evolutionary, ecological, and epidemiological success of high-risk antibiotic-resistant bacterial clones.

## Novel approaches to understanding the role of bacterial metabolism in colonization success

The systematic and complete understanding of how bacteria use nutrients within natural microbial communities is complicated by the cost, technical difficulty, and sensitivity of metabolomic studies to external perturbations (Johnson and Gonzalez 2012). Several new approaches have recently facilitated the study of microbial metabolism at an unprecedented scale. The following sections will delve into these new methods and discuss their potential to provide critical insights to combat and anticipate the emergence of the next pandemic high-risk clone.

### In silico predictive approaches: modeling metabolism

**Fig. 2:**
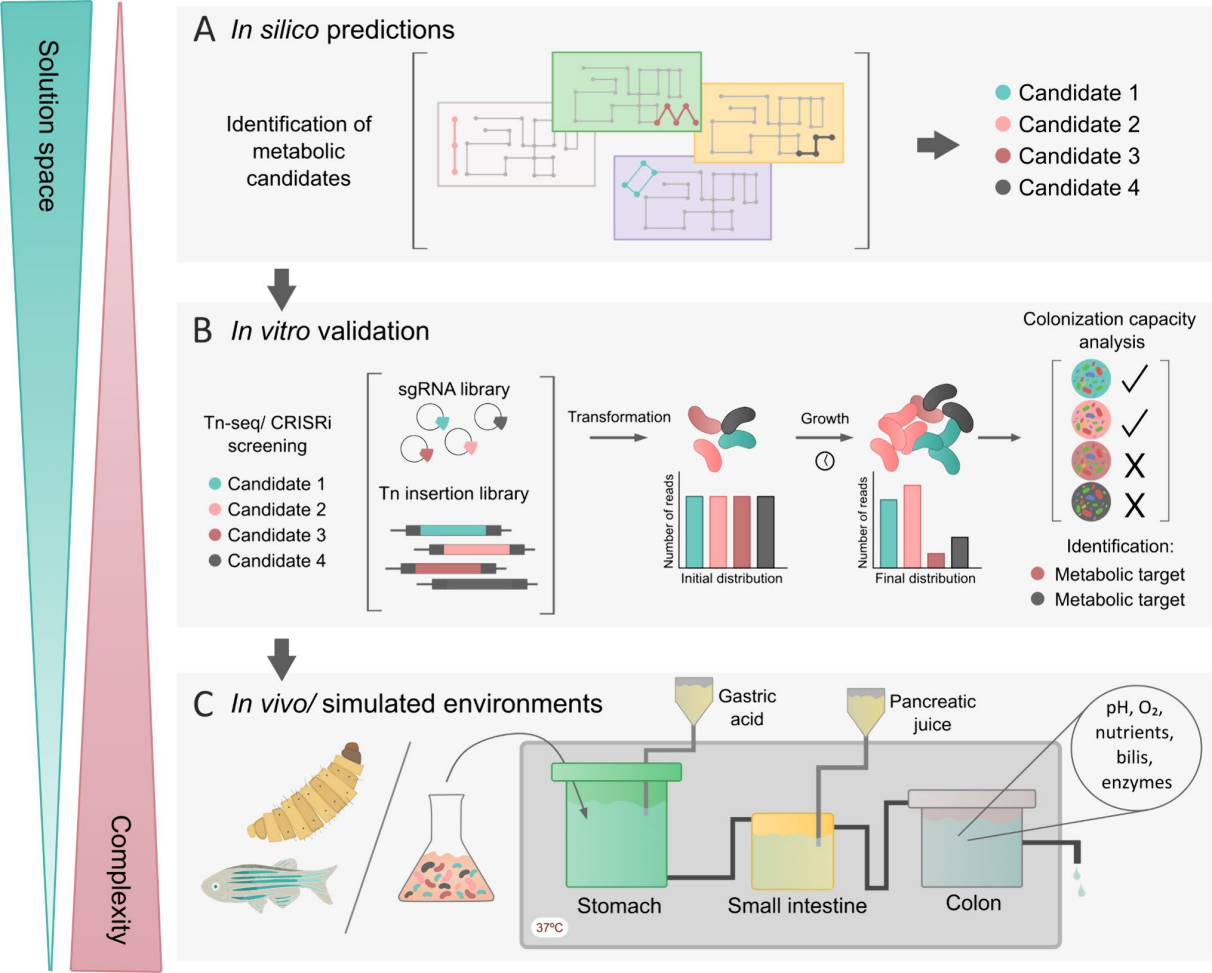
Overview of novel approaches to unravel the role of metabolic traits driving bacterial gut colonization. Wide large-scale computational analyses are followed by more targeted approaches that reduce the solution space, increasing the complexity of the resulting response. (**A**) Genome-scale metabolic models (GEMs) are structured representations of an organism's metabolic capabilities based on biochemical, genetic, and phenotypic knowledge, providing a map of all biochemical reactions within a cell. Comparing different GEM maps allows the exploration of potential metabolic candidates responsible for enhancing or preventing colonization. (**B**) Workflow for in vitro validation of the metabolic predictions obtained from GEMs. The fitness effect of any given mutant can be tracked using high-throughput genome sequencing techniques such as CRISPRi or Tn-Seq, which facilitate the validation process. Constructing arrayed libraries allows genome-wide metabolic interrogation and by comparing initial and final mutants’ abundance, factors associated with under-represented populations can be highlighted as metabolic targets. (**C**) Following phenotype confirmation, models that simulate the conditions of the gastrointestinal tract can be used to elucidate the role of metabolic traits in gut colonization. Non-classical animal models such as zebrafish or Galleria mellonella, together with mice models, constitute an approximation to test the targets’ performance in vivo but still miss some human gut inherent characteristics. Gastrointestinal simulators may overcome these drawbacks, mimicking intestinal conditions in different compartments and representing the different human intestinal sections to address the colonization capacity of the different species

The advent of sequencing technologies and a large amount of genomic high-throughput data have contributed to a profound understanding of microbial behavior at a systemic level (Bordbar et al. 2014). Genome-scale metabolic models (GEMs) are structured representations of the metabolic capabilities of a target organism based on existing biochemical, genetic, and phenotypic knowledge that can be used to predict phenotype from genotype (Nielsen 2017). In other words, GEMs map all the biochemical reactions that occur within a cell and can predict the growth of an organism in a given environment (Fig. 2A). GEMs are key biotechnology tools that are increasingly used to analyze and predict bacterial metabolism under different environmental and clinical conditions (Monk et al. 2014; O’Brien et al. 2015). For instance, GEMs of more than 100 *E. coli* strains suggested that the phylogroup B2 share a unique set of metabolic features: thanks to the presence of specific aldolases, B2 strains are more efficient than other *E. coli* phylogroups at metabolizing sugars derived from the mucus glycan. This confers a colonization advantage that might explain their outstanding gut colonization ability and epidemiological success (Fang et al. 2018).

The predictive power of individual strain GEMs is limited because bacterial strains do not live in isolation. Instead, they live in diverse communities where the extent of metabolic interactions has traditionally been difficult to understand (Ankrah et al. 2021). In this regard, the transition from single-taxon to community-scale metabolic models fostered by computational and mathematical advances has represented a paradigm shift in recent years (Harcombe et al. 2014; Zomorrodi and Segrè 2017; Dukovski et al. 2021; Woo et al. 2024). Community-scale models allow the understanding of complex microbial communities and their interspecies interactions, such as mutualism or competition (Lewis et al. 2012; Heinken et al. 2013; Ang et al. 2018; Dukovski et al. 2021; Schäfer et al. 2023; Ghiotto et al. 2024). Crucially, recent work has linked the composition of bacterial communities with its function, enabling the accurate prediction of biological function at the genetic, organismal, and ecological scales (Diaz-Colunga et al. 2023). This advance paves the way for a better understanding of gut microbiota ecological interactions and potentially engineering bacterial communities as therapeutic alternatives. In summary, (community-scale) GEMs are potent tools for elucidating the metabolism of single and complex systems and represent a great platform to study the metabolic capability associated with gut colonization of high-risk clones within the intricate environment of the gut microbiome (Heinken et al. 2023).

### In vitro arrayed perturbation experiments

Modeling helps to understand and predict the role of metabolic traits on gut colonization, yet experimental validation is crucial to confirm these predictions (Fig. 2B). Validating computational metabolic predictions may seem challenging, considering that GEMs generate extensive amounts of data. However, recent approaches offer accessible solutions. One such strategy involves the construction of genome-wide single-gene knockout mutant libraries, such as the KEIO collection for *E. coli* (Baba et al. 2006). This collection contains strains with mutations in each individual gene, allowing researchers to systematically assess the impact of each gene on a specific function. The use of the KEIO collection allowed the identification of 45 *E. coli* genes key for the inhibition of *P. aeruginosa* gut colonization in a nutrient-dependent manner (Christofi et al. 2019). This study highlights the power of genome-wide mutant libraries to uncover the ecological and evolutionary aspects that rule colonization.

However, genome-wide mutant libraries are only available for model bacteria and, therefore, are unsuitable for non-model species in the microbiota. Molecular biology has made significant progress in the dynamical engineering of cellular metabolism, especially with the development of CRISPR/Cas9 gene editing technology (Wang et al. 2016). Recent advances focus on using a catalytically dead version of Cas9 (dCas9) that cannot cleave DNA but retains a strong DNA binding activity. The binding of dCas9 to promoters and open reading frames efficiently prevents the expression of targeted genes by blocking transcription⁠. Hence, dCas9, combined with a single-guide RNA (sgRNA) targeting the chosen gene, can bind and repress targeted genes strongly and specifically (Bikard et al. 2013; Call and Andrews 2022). This system is called CRISPRi (for CRISPR interference) and has been validated under several experimental conditions and bacterial species (Bikard et al. 2013; Rousset and Bikard 2020; Call and Andrews 2022)⁠. By generating large sgRNA libraries redundantly targeting the whole genome of target bacteria, CRISPRi can be used to interrogate bacterial phenotypes in a high-throughput manner (Rousset and Bikard 2020), allowing the study of complex bacterial traits, such as colonization capacity, at an unprecedented resolution⁠. This technology enables the decryption of metabolic interactions by genetically engineering non-model bacterial species. For example, CRISPRi was used in the non-model probiotic strain *Eubacterium limosum* to study the role in gut colonization of several genes in the Wood-Ljungdahl pathway and the fructose-phosphotransferase system, paving the way for future metabolic studies in a non-traditionally studied bacteria (Shin et al. 2019; Call and Andrews 2022).

Transposon insertion sequencing (Tn-seq) offers another useful approach to uncovering complex metabolic interactions. This technique combines high-throughput DNA sequencing with transposon genome-wide mutagenesis. When a transposon is inserted in a gene, its function will be disrupted, hence revealing critical aspects of gene functionalities (Van Opijnen et al. 2009). Tn-seq requires generating a transposon library containing mutants with insertions in every non-essential gene. After growth in a given environment, the abundance of each specific insertion is retrieved by deep sequencing. Comparing each mutant’s abundance before and after growth provides quantitative information correlating phenotype with genotype (Van Opijnen et al. 2009; Burby et al. 2017). For instance, the analysis of nearly all *Vibrio cholerae* genes through Tn-seq revealed that bacteria lacking *vgrG3* gene, displayed a reduced colonization capacity. VgrG3 is part of the Type 6 secretion system (T6SS), a major virulence mechanism in Gram-negatives, and therefore, a deficiency in this secretion system will result in a compromised ability to compete with commensal bacteria (Fu et al. 2013).

In summary, high-throughput genomic disruption and interaction experiments hold great potential to decode host-microbiome associations, improve functional profiling of microbial genomes, and tackle complex biological pathways that underlie the colonization success of bacterial pathogens.

### In vivo and simulated environments

While the above-mentioned in silico and in vitro techniques provide a huge amount of information, they offer a limited view of gut colonization due to their static and simplified nature. In silico and in vitro techniques cannot fully replicate the dynamic interplay between the gut’s changing environment (pH, temperature, bile salts, etc.) and the complex biochemical processes and microbiota interactions within the human gastrointestinal tract (Morelli 2000).

Complex environments that mimic natural conditions should be employed to further validate insights identified through in silico and in vitro assays (Fig. 2C). Mice are arguably the most common animal model to study host-pathogen interactions (Wilk and Schughart 2012; Smith et al. 2022); however, ethical and cost-related issues preclude their use in many laboratories. Alternative model systems are progressively gaining importance. For instance, *Galleria mellonella* has been increasingly used as a model to study bacterial infection (Ménard et al. 2021), particularly by multi-resistant bacteria (Krezdorn et al. 2014). This invertebrate presents an immune system similar to mammals, and the research findings in *G. mellonella* often correlate with mammal results. In addition, the handling of the specimens is more straightforward than that of other invertebrate models. Vertebrate alternatives, such as Zebrafish (*Danio rerio*), also serve as models for studying host-microbiome interactions (Yang et al. 2022; Kaszab et al. 2023). Nevertheless, even the animal models listed above may not accurately capture some aspects of the complex interactions of the human gut.

Motivated by the complex, costly, and ethically regulated process associated with animal models (Dupont et al. 2019), novel approaches simulating intricate gut microbiota environments have emerged as promising alternatives. For example, human-associated gut microbiota microcosms are scalable and easy to manipulate in the laboratory while offering an ex vivo model of the human gut microbiota that partially preserves its natural diversity. In this model, anaerobic microcosms are filled with a fresh human fecal slurry that includes the resident microbial community. Human gut microbiota microcosms have been used to study colonization by high-risk clones (Benz et al. 2021) and horizontal gene transfer of antibiotic resistance genes (Baumgartner et al. 2020).

A more realistic yet complex model system is the gastrointestinal simulator. This in vitro platform dynamically mimics the human digestive processes (Dupont et al. 2019). Gastrointestinal simulators typically consist of a series of compartments that mimic the physiological conditions found in different gastrointestinal tract regions, such as the stomach, small intestine, and colon. Gastrointestinal simulators allow to experimentally dissect how digestion and nutrient availability along the gut influence human health and disease by simulating the complex interactions between food components, gut microbiota, and host cells. The efficacy of this approach has been demonstrated in studies investigating the potential survival and activity of probiotics in the gastrointestinal tract (Thuenemann 2015; Lambrecht et al. 2019; Liu et al. 2020). The use of gastrointestinal simulators provides a valuable and robust platform to inform in vivo experiments, reducing costs and animal use and aligning with recent European Directives promoting non-animal approaches for research (European Commission 2024). Remarkably, using human fecal samples to seed the simulator may help to study the metabolic ability and colonization capacity of high-risk clones in a real-life complex environment.

## Conclusions

While the gut microbiota composition varies significantly between individuals, recent findings suggest a functional convergence that transcends specific bacterial species (Diaz-Colunga et al. 2023). This implies that the metabolic capabilities of the gut microbiota, rather than its exact composition, play a critical role in enabling successful colonization by high-risk clones. Consequently, the future of gut microbiome research is moving beyond a species-centric view towards a functional understanding of the metabolic landscape. This shift will not only enhance our ability to combat antibiotic resistance but will also pave the way for developing novel therapeutic interventions and personalized medicine approaches based on an individual’s unique gut metabolic profile. In this regard, novel techniques like GEMs, CRISPRi, and Tn-Seq, combined with new in vitro and in vivo models that mimic gut conditions, will help to dissect the intricate metabolic interplay within the gut microbiome. Identifying the metabolic strengths and weaknesses of high-risk clones will be crucial to establishing targeted strategies to disrupt their colonization ability and predict the emergence of antibiotic-resistant threats.

## Data Availability

No datasets were generated or analyzed during the current study.
